# A decision support tool for the location, districting and dimensioning of Community Health Houses

**DOI:** 10.1007/s10729-025-09729-3

**Published:** 2025-11-10

**Authors:** Martina Doneda, Ettore Lanzarone, Carlotta Franchi, Sara Mandelli, Angelo Barbato, Alessandro Nobili, Giuliana Carello

**Affiliations:** 1 Department of Electronics, Information and Bioengineering, Politecnico di Milano, Milan, Italy; 2 National Research Council, Institute for Applied Mathematics and Information Technologies, Milan, Italy; 3 Department of Management, Information and Production Engineering, University of Bergamo, Dalmine (BG), Italy; 4 Department of Health Policy, Istituto di Ricerche Farmacologiche Mario Negri IRCCS, Milan, Italy

**Keywords:** Community Health Houses, Decision support system, Dimensioning, Integrated network planning, Location and districting, Operations research operations management

## Abstract

Community Health Houses (CHHs) are new entities in the Italian National Health Service that have been envisaged to provide proximity care to an increasingly aging population, and bear some similarities to other facilities in countries that have historically focused on public healthcare. This work proposes an integrated decision support system (DSS) for their planning, envisioned during the aftermath of the COVID-19 pandemic, which highlighted the frailty of the existing system. The DSS is based on an integer linear programming (ILP) model that simultaneously makes location, districting and dimensioning decisions for CHH, and accounts for accessibility and equity requirements. Based on Italian law yet designed in a parametrized way that makes it adaptable to several contexts, the DSS is able to design a hub and spoke network, which considers the provision of both mandatory and additional services. The sizes of the former are determined by directly taking into account the population served, while those of the latter are determined according to the specific demand for these services, accounting for diverse needs arising from different territories. The DSS also uses territorial units that refer to recognizable administrative areas. This ensures that the districting is easily recognized and accepted by the population. In addition to the ILP formulation, three decomposition-based matheuristics are proposed, which allow suitable solutions to be found within a reasonable time also for large and heterogeneous instances, while maintaining the flexibility of the ILP formulation. Computational results on synthetic realistic instances validated the DSS, while its application to a real-life case in a Northern Italian province demonstrated the effectiveness of the heuristic approaches and provided a proof of concept for its practical application.

**Supplementary Information:**

The online version contains supplementary material available at 10.1007/s10729-025-09729-3.

## Highlights

Decision support tool that quantitatively supports the design of an effective Community Health House (CHH) network

Simultaneous location, districting and dimensioning decisions for CHHs.

Novel ILP formulation with hub and spoke CHHs together, both mandatory and additional services, accessibility and equity requirements.

Three constructive decomposition-based matheuristics to find good solutions in reasonable time while keeping the flexibility of the ILP formulation.

Computational results on realistic instances validated the adequacy of the tool and the effectiveness of the matheuristic approaches.

**Supplementary Information:**

The online version contains supplementary material available at 10.1007/s10729-025-09729-3.

## Introduction

Healthcare systems are facing new challenges, and will likely encounter even more in the near future. On the one hand, due to an aging population, healthcare systems in Western countries are having to cope with an increased number of patients with age-related chronic conditions, in such volumes that fewer and fewer patients can be cared for in hospital settings. Consequently, considerable effort is needed to relieve the pressure on hospitals by enhancing prevention and proximity care. On the other hand, the COVID-19 pandemic exposed many structural weaknesses in the current primary care network of several countries, especially in industrialized countries with an aging population. In all of those countries, increased life expectancy has also led to an increased burden of morbidity [10].

Decision-makers must consider that this demographic trend is constant and irreversible and that pandemics or other crisis situations are likely to reoccur in the future. Therefore, the reorganization of health service delivery through the integration of community services and hospitals, to shift care and treatment away from hospitals as much as possible, is a necessity for the survival of national and regional health systems. In addition to reducing the burden on hospitals, so that they can focus on acute patients and treatments that require hospitalization, such reorganization could also improve the health and quality of life of patients, as it would allow the provision of timely and continuous care and increase the effectiveness of screening and prevention campaigns [9].

The COVID-19 pandemic hit Italy particularly hard, clearly accentuating the structural weaknesses of the primary care network, which was already struggling with overcrowded emergency departments and long waiting lists [4, 43]. Although the Italian National Health Service is generally considered to be capable of very high standards [18, 40], the pandemic highlighted the lack of coordination between primary care and territorial services, the fragmentation of care pathways, the underdevelopment of digital healthcare delivery alternatives, and the shortage of medical personnel possibly related to insufficient strategic resource planning [44]. For example, a surge of inappropriate emergency admissions in the acute care network has been observed in recent years, most of which were related to patients with minor illnesses who would have been more appropriately treated in primary care facilities.

One of the main causes attributed to this phenomenon is that obtaining care is often difficult for patients due to the limited opening hours of general practitioner (GP) offices and/or significant geographical distances [6].

To overcome these issues, the Italian National Recovery and Resilience Plan (INRRP), which is the national implementation of the European *NextGenerationEU* scheme, proposed a reorganization of the community health system spread throughout the country [33]. One of the main instruments of this reorganization are the so-called Community Health Houses (CHHs). They are meant to physically house GPs, pediatricians and other medical professionals in the same place to enhance interprofessional communication and leverage economies of scale for the deployment of more advanced technological solutions, e.g., telemedicine. This organizational innovation is particularly important for chronic patients, who make multiple appointments and visits to manage their condition. Some entities with characteristics similar to those envisioned for CHHs were already present in some Italian regions but with more limited functions, and they are now set to become an integral part of the CHH system [38]. With an aging population and a shortage of medical personnel, CHHs introduce a new organizational model that could play a key role in stabilizing the primary care system within the Italian National Health Service, thus redefining the functions of all the actors involved and coordinating them as necessary [11].

The Italian plan for CHHs is not unique in the international arena, as similar frameworks that focus on integrating GPs with specialist services and territorial proximity can be found elsewhere, particularly in countries with an historical focus on public healthcare; for example see the Brazilian Health Centers (*Centros de Saúde*) [3], the Slovenian Community Health Centers (*Zdravstveni Domovi*) [26], the Greek Local Health Units (*Topikes Monades Ygias*) [37] and the Australian *Community Health Centres* [45]. In particular, Italian CHHs share many similarities with the Greek system, which was envisaged with a cost rationalization approach after the 2008 financial crisis [37] under the EU Economic Adjustment Program. Other examples, albeit less similar, are the Latin American Local Health Systems (*Sistemas Locales de Salud*, SILOS), proposed by the Pan American Health Organization in the 1990s [39], and the private for-profit US Integrated Delivery Systems and Health Maintenance Organizations, e.g., the *Kaiser Permanente* [27]. Many of these systems are based on a hub and spoke model, which is a paradigm derived from air-traffic routing, wherein a main airport is used as a central point (a *hub*) for the coordination of flights to and from smaller airports, referred to as *spokes*. Also in logistics, goods can be sent first to a central warehouse (hub) and then distributed to smaller shops (spokes).

Implementing a CHH network and integrating it with the existing healthcare system requires making many long-term (where to locate the CHHs, how to allocate resources, in which facility the patients will be treated, etc.) to short-term (appointment scheduling, staff rostering, etc.) decisions. In this work, we focus on the long-term decisions concerning positioning, resource assignment and districting of CHHs, while considering the Italian requirements prescribed by law. Here, we define *districting* as the assignment of a set of territories to a CHH to create contiguous service areas. As the associated guidelines and laws are not all quantifiable statements, our first contribution to the literature is to formalize this problem and represent qualitative requirements in a quantitative way, so to formulate it through an integer linear programming (ILP) model [12]. Moreover, as it was found out to be computationally demanding or even intractable for realistic instances, our second contribution is the development of a set of problem-specific heuristics i.e., algorithms used for speeding up problem-solving when an exact approach is either too slow or when it cannot provide any feasible solution in a search space [32].

Our ultimate goal is to provide decision-makers with a DSS that can be used for CHH dimensioning and management. We made this DSS as generalizable as possible, building a parametrized structure that can be easily reconfigured to meet the needs of different territories and populations within the contexts of any public healthcare service in which primary care is provided outside of hospitals and secondary care can (at least partially) be provided in the same facility.

The rest of the paper is organized as follows: Section 2 describes the setting that has been considered and the modeling choices that lead from the qualitative guidelines to their mathematical representation. The literature on similar problems is reviewed in Section 3. Next, Sections 4 and 5 describe the problem formulation and the heuristic approaches, respectively. Finally, Section 6 presents the computational tests, and Section 7 concludes the paper with a summary of the contribution, the results and some suggestions for future research.

## Problem description

We consider the CHH setting, purpose and requirements as defined by the laws passed by the Italian government [35] within the INRRP framework [8].

The Italian law defines two types of CHHs, i.e., *hub* and *spoke* CHH, based on the hub and spoke organizational model. This model originated in the transportation industry – specifically in aviation – but has also proven efficient in other sectors, such as retail, education and healthcare [15]. Both hub and spoke CHHs are intended to provide primary and nursing care but differ with respect to the services they provide and the size of their reference population. Hub CHHs are intended to be the reference structure for urgencies and provide 24/7 care, thus addressing the demand of patients who need to be treated at night time or on weekends but do not present severe enough conditions to warrant hospital admission. GPs may be present in hub CHHs as well. In contrast, spoke CHHs are not open 24/7 and are primarily intended to provide GP services.

The Italian law also defines the reference population for both types of CHHs, and that of hub CHHs for 24/7 urgency services. As it is the case in classic hub and spoke models, hub CHHs are usually larger than spoke CHHs, i.e., the reference population for a hub CHH is between 40’000 and 50’000 people, while that for a spoke CHH is 35’000 at most. Moreover, the reference population for 24/7 urgency services in a hub CHH is 100’000.

In addition, one of the purposes of CHHs is to provide, on a territorial basis, *specialist care services for high-prevalence diseases*, which are currently provided by hospitals. Therefore, in addition to 24/7 care and GPs’ offices, CHHs need to house other functions, such as specialized outpatient clinics and diagnostic services and, ideally, equipment for long-term/maintenance treatments that do not require hospitalization (e.g., physiotherapy). The resources allocated to CHHs for these services should depend on actual demand, and not just the size of the target population. The services provided by each facility (capability assignment) and the resources allocated (capacity dimensioning) must be decided, as well.

These data are listed in Table 1.

**Table 1:** Services provided by the two types of CHHs [35]

	Hub CHH	Spoke CHH
Primary care services	$\checkmark$	$\checkmark$
Nursing services	$\checkmark$	$\checkmark$
Specialist care services for high-prevalence pathologies	$\checkmark$	$\checkmark$
On-call personnel for 24/7 urgencies	$\checkmark$	– –
Reference population	40’000 – 50’000	$\le$ 35’000
Reference population for urgencies	100’000	– –

We address the problem of locating hub and spoke CHHs in a given territory that has been divided into basic territorial units that correspond to recognizable administrative areas, so that the districting would follow subdivisions that would be easily recognized and accepted by the population. Equity and accessibility are mandated by the Italian law, in the form of qualitative requirements. We assume that the fundamental issues in enforcing them are traveling time and distance, and we represent these requirements by forcing a maximum acceptable traveling time (coverage constraints) and closest assignment constraints.

## Related literature

CHHs are new entities in the Italian context^1^, and, to the best of our knowledge, this is the first time they have been discussed in a management science or operations research article, although a few papers have been published on related policies [31].

The primary problem that needs addressing when designing a CHH network is a type of location problem. Location problems, and discrete location problems (DLPs) in particular, have been widely studied in the literature [14], with many works describing applications in healthcare management as well [2].

However, it presents numerous different features and characteristics, such as different types of services, service dimensioning and coverage and proximity requirements, that have rarely been considered together in a single location problem. Most of the literature on healthcare-related DLPs focuses on hospitals, which have a completely different set of requirements and challenges than CHHs. In relation to CHHs, Khodaparasti et al. [28] designed a location model for a hierarchical system of community-based organizations (CBOs) in Iran that bridges the gap between communities and primary healthcare providers by offering, for example, counseling and addiction prevention services. However, they address a strongly hierarchical structure, in which CBOs act as an intermediate level between the population and more traditional primary care providers, thus differing from the CHH hub and spoke system. A more similar setting to CHHs can be found in the problem of locating perinatal facilities [19] or health centers [22, 36]. The literature on the planning of primary care network in rural settings is also relevant, as the geography of the Italian territory poses some challenges in this regard e.g., the coexistence of remote communities in the Alpine region and dense metropolitan areas [46].

In general, the degree of penetration of primary care services, particularly those aimed at providing preventive and social services, has been found to strongly correlate with geographic proximity [5, 21], as accessibility is of paramount importance when designing a primary care system. Perhaps the most prominent research work on this topic in recent years is that by Weiss et al. [49], in which the authors map the time required to reach the closest healthcare facility from each point on Earth either using a motorized vehicle or on foot. A very interesting finding from their study is that only $\sim 9\%$ of the world’s population cannot reach a healthcare facility within an hour if they have access to a private vehicle, but this number rises to $\sim 43\%$ if they can only walk. Unfortunately, the study did not provide data on travel time for public transportation.

Therefore, a vital component of any CHH planning tool is the proximity requirement, which results in closest assignment constraints. Closest assignment is a fundamental requirement in healthcare systems other than CHH. Mestre et al. [34] implemented two location-allocation models to address uncertainty in long-term hospital network planning, imposing that demand is to be met by the closest facility that can satisfy that type of demand. In contrast, Acar and Kaya [1] considered the design of a mobile healthcare network for disasters such as earthquakes. They compared two models: one in which allocation to mobile hospitals was determined by a central agency and one in which demand was allocated using a closest assignment constraint. They demonstrated that, although travel costs decreased through the use of such constraints, the total cost generally increased due to the resulting imbalance among hospitals. Although they did not enforce the closest assignment constraint, Gu et al. [23] also considered distance to be of primary importance for service participation. They presented an optimization method to solve the preventive healthcare facility location problem. Similar to the CHH problem, they considered a minimum number of *clients* required to keep the facility operational. In contrast to our geography-based allocation, they assumed that people would choose their target location and did not use closest assignment constraints.

The problem of CHH network design is also closely related to the problem of district design. Farughi et al. [17] proposed a new multi-objective mathematical model for the designing of compact, balanced and contiguous healthcare districts.

As mentioned, the design of the CHH network shares several features with facility location problems, but with some distinctive features, summarized as follows. There are two main types of facilities, hub and spoke, but services provided to patients can be considered as types of facilities on their own, each with a unique capability. Assignment is required individually for each service, and each territorial unit can be served by a limited number of different CHHs. Facilities are capacitated, some are defined by both a capacity and a threshold, and some services are dimensioned in a modular way. Coverage and closest assignment constraints are considered while minimizing opening and service costs. Each of these features alone can have a significant impact on the problem, and combining different features can greatly change the computational effort needed to solve the problem. For instance, although closest assignment is automatically guaranteed in some problems with distance-based objective function such as the *p*-median, single and closest assignment are not always guaranteed [20]. If capacity is considered, the closest assignment constraint must be explicitly enforced, as it occurs for the problem considered here. Problems with both capacity and closest assignment constraints are not common. Although some research works have described problems similar to the one of designing a CHH network, they do not consider all the above-listed key features included in it. For example, in Teixeira et al. [47], a hierarchical facility location model for the public sector is presented, and it does indeed combine capacity and closest and single assignment constraints; in this work, facilities can be opened or closed, and different services are considered, but opening and dimensioning costs are not considered; moreover a *p*-median-like model is applied to a secondary school network; along with the different application sector, only one type of service is considered. In Çalık et al. [13], closest assignment is considered so as to guarantee reasonable and fair traveling time for the participants to geographically-distributed exams; only one type of facility is considered. Finally, the problem addressed in Ko et al. [29] shares many features with the CHH problem: choice of facility and of their capabilities, capacity dimensioning with an upper limit, different types of diseases/services, closest assignment; however, coverage constraint are not considered and a demand point can be served by any number of sites per service.

## Problem representation and ILP formulation

We represent the considered territory as a set *I* of basic territorial units, with the population of each denoted by $p_i$, and demands for various health services. The units represent individual cities or their groupings of limited size; in the case of large cities, they represent a neighborhood with a limited number of inhabitants. The entire population of each unit $i \in I$ has to be necessarily assigned to the same CHH for the same service. The set *I* represents not only the territorial units along with their demand but also the set of candidate sites for CHHs. In other words, every unit *i* is characterized by its own population and demand for services, and at the same time, it can also host a CHH.

**Fig. 1:**
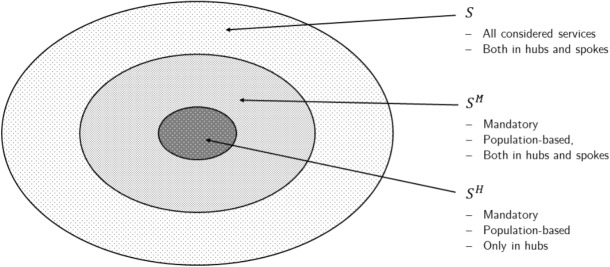
Hierarchy of the services provided by CHH

We described the services provided by CHHs through the set *S* of the different services. It is structured as illustrated in Fig. 1 and it includes mandatory (denoted by $S^M \subset S$) and additional services (represented by $S \setminus S^M$). For example, mandatory services include GP care and nurse assistance. Some of the mandatory services can be provided by both hub and spoke CHHs, while hub services $S^H \subset S^M$ can only be provided by hub CHHs. The most important service in $S^H$ is the 24/7 assistance for urgencies.

The mandatory services are sized directly taking into account the population served, while the dimensioning of the additional services takes into account the specific demand for these services in the units. For population-based services, parameters $l^H_s$ and $l^M_s$ denote the maximum population to which a CHH can provide a service $s \in S^H$ or $\in S^M \setminus S^H$. In addition, the population that can be assigned to a hub CHH must lay between a quantity $b^{min}$ and $b^{max}$, as defined by law. As for demand-based services, these include, for example, specialist visits, diagnostic examinations and physiotherapy. For these, $d_{si}$ denotes the demand for a service $s \in S$ in unit $i \in I$.

CHHs need to be located close to the communities they serve. We represent this requirement by establishing a maximum travel time between each unit and the reference CHH and taking into account known travel times $t_{ij}$ between any two units $i \in I$ and $j \in I$. Here, $\zeta _i^H$ and $\zeta _i$ denote the maximum travel time from unit $i \in I$ to either the hub or the spoke CHH to which *i* is assigned, respectively. Furthermore, within these maximum travel times, we impose that each unit $i \in I$ be assigned for each service $s \in S$ to the closest CHH providing *s*. $\zeta _i^H$ and $\zeta _i$ thresholds, together with $b^{\max }$, ensure fair access in both urban and rural settings. The upper limit of the reference population prevents any single CHH from serving too many people in densely populated areas, avoiding overcrowding. Conversely, appropriate travel time thresholds ensure that each community has access to the CHH network within a reasonable distance, even in sparsely populated areas.

Finally, to ensure that the districting can be applied in practice, the number of CHHs to which each unit can be assigned for the various services is limited by parameter $\eta$.

The districting choices are centralized, and, therefore, assignment choices do not take user preferences into account. However, CHHs also host GPs , and the Italian law states that users are forced to choose one GP from those assigned to the territory in which they live. Moreover, chronic patients, who make multiple visits, may benefit from some of the specialists treating them being in the same physical location as their GP and, by always referring to the same CHH, the continuity of care provided to them can be improved. Therefore, it is expected that patients will mostly choose their own referral center, and this makes it reasonable to assume centralized assignments.

There are both strategic and operational costs associated with CHHs. The strategic cost of creating a new CHH includes that of constructing a new building or renovating an existing building to house the CHH as well as that of general equipment. We denote the cost of opening a hub and a spoke CHH, using $k^H$ and $k^S$, respectively. Moreover, the strategic cost of activating a new service $s \in S$ in a CHH, which includes the costs of the specific equipment and devices required for delivering it, is denoted by $f_s$. Further, the operating cost of delivering service $s \in S$ is associated with the shifts of the personnel trained to provide that service, whose cost per shift of service $s \in S$ is denoted by $c_s$. Finally, the number of individual exams, visits or treatments for service $s \in S$ deliverable in a single shift, which converts working shifts into served demand $d_{si}$, is denoted by $\alpha _s$.

CHHs location decisions are modeled with binary variables $y_i$, equal to 1 if a CHH is opened in unit $i \in I$, and 0 otherwise. Additional binary variables $y_i^H$ and $y_i^S$ represent the choice between opening a hub or spoke CHH, respectively: a CHH opened in $i \in I$ can be either a hub ($y_i^H = 1$) or a spoke ($y_i^S = 1$). The services provided by a CHH opened in $i \in I$ are defined using binary variables $z_{is}$, which are equal to 1 if the CHH located in $i \in I$ provides service $s \in S$. Further, the districting is represented by binary variables $x_{ijs}$, which are equal to 1 if unit $j \in I$ is served by a CHH opened in $i \in I$ for service $s \in S$. Integer non-negative variables $w_{is}$ represent the number of shifts of a health professional trained in service $s \in S$ at the CHH located in unit $i \in I$. Finally, binary variables $\chi _{ij}$ are equal to 1 if unit $j \in I$ is served by CHH $i \in I$ for at least one service.

Several decision-making levels, both in a hierarchical and a temporal sense, are considered simultaneously in this setting. Although it may seem unrealistic, we argue that, in such an interconnected system, a rigid sequence of decisions may lead to the implementation of partial solutions that, although optimal at their respective decision-making level, can result in suboptimal configurations at lower decision-making levels. To confirm this insight, one of the proposed heuristics decomposes the problem into sequential decisions, offering the possibility of evaluating the effect of such an approach.

The corresponding sets, parameters and decision variables are summarized in Table 2.

**Table 2:** Sets, parameters and decision variables of the ILP model

Sets	
*I*	territorial units
*S*	services
$S^M \subset S$	mandatory services
$S^H \subset S^M$	mandatory services that can be provided only at hub CHH
Parameters	
$b^{min}$	minimum reference population for a hub CHH
$b^{max}$	maximum reference population for a hub CHH
$t_{ij}$	travel time from $i \in I$ to $j \in I$
$\zeta _i$	maximum travel time for reaching a CHH from unit $i \in I$
$\zeta ^H_i$	maximum travel time for reaching a hub CHH from unit $i \in I$
$l^H_s$	maximum number of people served in a hub CHH for service $s \in S^H$
$l^M_s$	maximum number of people served in a CHH for service $s \in S^M \setminus S^H$
$p_i$	population of $i \in I$
$d_{si}$	demand for $s \in S$ from $i \in I$
$\alpha _s$	number of interventions for service $s \in S$ deliverable in a shift
$k^H$	fixed cost for opening a hub CHH
$k^S$	fixed cost for opening a spoke CHH
$f_s$	fixed cost for activating service $s \in S$
$c_s$	cost of one shift for service $s \in S$
$\eta$	maximum number of CHHs to which a unit can be assigned
Decision variables	
$y_i \in \{0,1\}$	opening of a CHH in $i \in I$
$y_i^H \in \{0,1\}$	opening of a hub CHH in $i \in I$
$y_i^S \in \{0,1\}$	opening of a spoke CHH in $i \in I$
$z_{is} \in \{0,1\}$	activation of service $s \in S$ in CHH located in $i \in I$
$w_{is} \in \mathbb {N}$	number of shifts for service $s \in S$ activated at the CHH
	located in $i \in I$
$x_{ijs} \in \{0,1\}$	allocation of unit $j \in I$ to CHH located in $i \in I$ for service $s \in S$
$\chi _{ij} \in \{0,1\}$	equal to 1 if unit $j \in I$ is assigned to a CHH located in $i \in I$
	for at least one service

The first set of constraints forces the opening of CHHs and the activation of services in them:

1 $$\begin{aligned}&y_i^H + y_i^S = y_i&\forall i \in I \end{aligned}$$

2 $$\begin{aligned}&y_i^H \ge z_{is}&\forall i \in I, s \in S^H \end{aligned}$$

3 $$\begin{aligned}&y_i \ge z_{is}&\forall i \in I, s \in S \setminus S^H \end{aligned}$$

4 $$\begin{aligned}&\left\lceil {\frac{\sum _{j \in I}p_j}{b^{min}}} \right\rceil \ge \sum _{i \in I} y_i^H \ge \left\lceil {\frac{\sum _{j \in I}p_j}{b^{max}}} \right\rceil \end{aligned}$$

Constraints (1) state that each CHH is either a hub or a spoke. Constraints (2) and (3) guarantee that services are provided only at opened CHH: Constraints (2) refer to hub services $s \in S^H$, while Constraints (3) refers to all services $s \in S \setminus S^H$. Constraint (4) bounds the number of hub CHHs that are opened based on the total population.

The second set of constraints guarantees that the selected services are provided to each unit and that each unit is assigned to a close enough CHH:

5 $$\begin{aligned}&\sum _{i \in I: t_{ij} \le \zeta _{j}^H} y_i^H \ge 1&\forall j \in I \end{aligned}$$

6 $$\begin{aligned}&\sum _{i \in I: t_{ij} \le \zeta _j} y_i \ge 1&\forall j \in I \end{aligned}$$

7 $$\begin{aligned}&\sum _{i \in I: t_{ij} \le \zeta _j^H} x_{ijs} = 1&\forall j \in I, s \in S^H \end{aligned}$$

8 $$\begin{aligned}&\sum _{i \in I: t_{ij} \le \zeta _j} x_{ijs} = 1&\forall j \in I, s \in S \setminus S^H \end{aligned}$$

Constraints (5) and (6) ensure that at least one hub and one CHH, respectively, is opened close enough to each unit. Constraints (7) and (8) guarantee that the demand for each service at any site $j \in I$ is met by one CHH, distinguishing between $S^H$ and $S \setminus S^H$.

The third set of constraints ensures the closest assignments:

9 $$\begin{aligned}&z_{is} + \sum _{h \in I; t_{ij} < t_{hj}} x_{hjs} \le 1&\forall i \in I, j \in I, s \in S^H, \nonumber \\ & t_{ij} \le \zeta _j^H, t_{hj} \le \zeta _j^H \end{aligned}$$

10 $$\begin{aligned}&z_{is} + \sum _{h \in I; t_{ij} < t_{hj}} x_{hjs} \le 1&\forall i \in I, j \in I, s \in S \setminus S^H, \nonumber \\ & t_{ij} \le \zeta _j, t_{hj} \le \zeta _j \end{aligned}$$

They guarantee that each unit is served by the closest available and suitable CHH, either by a hub CHH (9) or any CHH (10), for all required services. They state that, if a service $s \in S$ is activated in $i \in I$, each territorial unit $j \in I$ cannot be assigned, as far as *s* is concerned, to a CHH farther than the one located in $i \in I$ (formulation of the closest assignment constraint first proposed in [48], see [16] for possible alternative formulations).

The fourth set of constraints guarantees that a unit is assigned to a CHH within the maximum travel time to receive a service only if that service is activated at the CHH:

11 $$\begin{aligned}&x_{ijs} \le z_{is}&\forall i \in I, j \in I, s \in S^H: t_{ij} \le \zeta _j^H \end{aligned}$$

12 $$\begin{aligned}&x_{ijs} \le z_{is}&\forall i \in I, j \in I, s \in S \setminus S^H: t_{ij} \le \zeta _j \end{aligned}$$

Constraints (11) refer to $S^H$, while Constraints (12) refers to $S \setminus S^H$.

The fifth set contains the capacity constraints:

13 $$\begin{aligned}&\sum _{j \in I: t_{ij} \le \zeta _j^H} p_j x_{ijs} \le l^H_s y_i^H&\forall i \in I, s \in S^H \end{aligned}$$

14 $$\begin{aligned}&\sum _{j \in I: t_{ij} \le \zeta _j }p_j x_{ijs} \le l^M_s y_i + \left( l^H_s - l^M_s \right) y_i^H&\forall i \in I, s \in S^M \setminus S^H \end{aligned}$$

15 $$\begin{aligned}&\sum _{j \in I: t_{ij} \le \zeta _j} d_{js} x_{ijs} \le \alpha _s\cdot w_{is}&\forall i \in I, s \in S \setminus S^M \end{aligned}$$

Constraints (13) and (14) guarantee that the capacity of hub and spoke CHHs, respectively, is not exceeded by the volume of population assigned to them. Constraints (15) guarantee, for each service $s \in S \setminus S^M$, that the number of shifts in any open CHH is enough for meeting all the assigned demand.

The sixth set of constraints sets the maximum number of CHH to which each unit can refer to:

16 $$\begin{aligned}&\left\lceil {\frac{\sum _{j \in I }p_j}{b^{min}}} \right\rceil \chi _{ij} \ge \sum _{s \in S: t_{ij} \le \zeta _j^H} x_{ijs}&\forall i \in I, j \in I: t_{ij} \le \zeta _j \end{aligned}$$

17 $$\begin{aligned}&\sum _{i \in I: t_{ij} \le \zeta _j^H}\chi _{ij} \le \eta&\forall j \in I \end{aligned}$$

Finally, Constraints (18) to (21) define the domains of the decision variables:

18 $$\begin{aligned}&y_i, y_i^H, y_i^S \in \{0,1\}&\forall i \in I \end{aligned}$$

19 $$\begin{aligned}&z_{is} \in \{0,1\}, w_{is} \in \mathbb {N}&\forall i \in I, s \in S \end{aligned}$$

20 $$\begin{aligned}&x_{ijs} \in \{0,1\}&\forall i \in I, j\in I, s \in S \end{aligned}$$

21 $$\begin{aligned}&\chi _{ij} \in \{0,1\}&\forall i \in I, j \in I \end{aligned}$$

The objective function (22) is the sum of all the costs of the designed network, including opening, service activation and operating costs:

22 $$\begin{aligned} \sum _{i \in I} \left( k_i^H y_i^H + k_i^S y_i^S \right) + \sum _{i \in I}\sum _{s\in S} \left( f_s z_{is} + c_s w_{is} \right) \end{aligned}$$

Therefore, the exact formulation, denoted as $\mathcal {P}$, is as follows:

$$\begin{aligned} \mathcal {P}: \left\{ \begin{array}{l} \min \; (22) \\ \text {s.t.} \\ \quad (1) - (21) \end{array} \right. \end{aligned}$$

## Heuristic approaches

Preliminary tests showed that solving the exact formulation $\mathcal {P}$ to optimality can be by $k^H$ and $k^S$ extremely computationally demanding for instances of realistic size. Even for large and heterogeneous instances, we could not obtain an integer solution within one hour. Therefore, we propose three heuristic approaches based on constructive decomposition-based matheuristics (Section 5.1), which allowed us to obtain good solutions in a reasonable amount of time while maintaining the flexibility of the ILP formulation. Moreover, the heuristic solutions can be refined, as presented in Section 5.2.

### Constructive heuristics

The first constructive heuristic ($H_1$), described in Algorithm 1, is based on a hierarchical decomposition of $\mathcal {P}$ into three successive problems, which follows the importance of the decisions in this problem. They are described by the same number of ILP formulations:

1. $\mathcal {P}^H$, which considers only hub CHH and services in $S^H$:
  $$\begin{aligned} \mathcal {P}^H: \left\{ \begin{array}{l} \min \; (22) \\ \text {s.t.} \\ \quad (1), (2), (4), (5), (7), (9), (11), (13), (18)-(21) \end{array} \right. \end{aligned}$$
  It solves the problem of locating the hub CHH and assigning the services $s \in S^H$ to them. The optimal choices are stored in parameters $\hat{y}_i^H$ and $\hat{z}_{is}$.
2. $\mathcal {P}^M$, which considers all mandatory services:
  $$\begin{aligned} \mathcal {P}^M: \left\{ \begin{array}{l} \min \; (22) \\ \text {s.t.} \\ \quad (1), (2), (4), (5)-(7), (9), (11), (13), \\ \quad \quad \quad (14), (16), (17), (18)-(21) \\ \quad y_i \ge z_{is} \quad \forall i \in I, s \in S^M \setminus S^H \\ \quad \sum _{i \in I: t_{ij} \le \zeta _j} x_{ijs} = 1 \quad \forall j \in I, s \in S^M \setminus S^H \\ \quad z_{is} + \sum _{h \in I: t_{ij} < t_{hj}}x_{hjs} \le 1 \quad \forall i \in I, j \in I, s \in S^M \setminus S^H: \\ \hspace{5.2cm} t_{ij} \le \zeta _j, t_{hj} \le \zeta _j \\ \quad x_{ijs} \le z_{is} \quad \forall i \in I, j \in I, s \in S^M \setminus S^H: t_{ij} \le \zeta _j \\ \quad y_i^H \ge \hat{y}^H_i \qquad \forall i \in I \\ \quad z_{is} \le \hat{z}_{is} \qquad \forall s \in S^H \end{array} \right. \end{aligned}$$
  Here, the explicitly written constraints correspond to Constraints (3), (10) and (12) of $\mathcal {P}$, but they are restricted to the mandatory services that can be provided by any CHH, i.e., $s \in S^M \setminus S^H$. Solving $\mathcal {P}^M$ selects the positions of spoke CHHs and, possibly, additional hub CHHs and assigns the mandatory services to the CHH while retaining those selected by $\mathcal {P}^H$ ($y_i^H \ge \hat{y}^H_i$) and the hub services assignment ($x_{ijs} \le z_{is}$, $z_{is} \le \hat{z}_{is}$). The optimal choices are stored in parameters $\hat{y}_i^H$, $\hat{y}_i^S$ and $\hat{z}_{is}$.
3. $\mathcal {P}_1$, which completes the solution:
  $$\begin{aligned} \mathcal {P}_1: \left\{ \begin{array}{l} \min \; (22) \\ \text {s.t.} \\ \quad (1) - (21) \\ \quad y_i^H \le \hat{y}^H_i \qquad \forall i \in I \\ \quad y_i^S \le \hat{y}^S_i \qquad \forall i \in I \\ \quad z_{is} \le \hat{z}^H_i \qquad \forall i \in I, s \in S^M \end{array} \right. \end{aligned}$$
  This includes $\mathcal {P}$ and additional constraints. Constraints $y_i^H \le \hat{y}^H_i$ and $y_i^S \le \hat{y}^S_i$ restrict the location of hub and spoke CHHs to those selected by $\mathcal {P}^H$ and $\mathcal {P}^M$; likewise, Constraints $z_{is} \le \hat{z}^H_i$ restricts service assignments.

**Algorithm 1:**
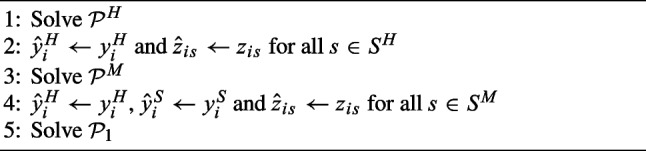
Heuristic $$H_1$$.

The other constructive heuristics ($H_2$ and $H_3$), described in Algorithms 2 and 3, respectively, are based on a geographical decomposition. First, they solve $\mathcal {P}$ for subdivisions of the entire territory the only requirement for which is to be connected and contiguous. Since these divisions are smaller than the entire territory, $\mathcal {P}$ can be solved to optimality in a reasonable amount of time. However, the solution obtained by combining those for the individual territories cannot be immediately used to produce a feasible solution because of the closest assignment constraints and border effects. The two heuristics $H_2$ and $H_3$ differ in how feasibility is restored from the solutions in the subdivisions.

**Algorithm 2:**
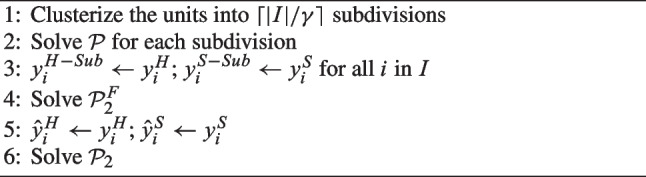
Heuristic $$H_2$$.

**Algorithm 3:**
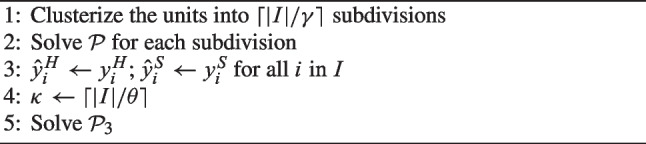
Heuristic $$H_3$$.

Both heuristics $H_2$ and $H_3$ begin with the division of the |*I*| units into subdivisions using a *k*-means algorithm [30]. This algorithm allows for continuous subdivisions by construction without further post-processing. The number of clusters is fixed to $\lceil |I|/\gamma \rceil$, where $\gamma$ describes the ideal dimension of the subdivisions. Then, $\mathcal {P}$ is solved for each subdivision, in which the number of CHHs is once again determined by its population, based on Constraints (4). The resulting locations of hub and spoke CHHs in the subdivisions are stored in parameters $y_i^{H-Sub}$ and $y_i^{S-Sub}$, respectively. Finally, the solutions are merged while seeking feasibility.

In $H_2$, feasibility is also sought by allowing additional hub and spoke CHHs, but this is done by minimizing the number of differences with respect to the set of CHHs determined by the solutions obtained in each subdivision. Therefore, the solutions are merged by solving problem $\mathcal {P}^F_2$:

$$\begin{aligned} \mathcal {P}^F_2: \left\{ \begin{array}{l} \min \; \sum _{i \in I} \lambda _i \\ \text {s.t.} \\ \quad (1)-(21) \\ \quad y_i^H \le y^{H-Sub}_i + \lambda _i \qquad \forall i \in I \\ \quad y_i^S \le y^{S-Sub}_i + \lambda _i \qquad \forall i \in I \end{array} \right. \end{aligned}$$

Variables $\lambda _i$ represent the changes in the locations of CHHs with respect to those obtained for the subdivisions and are minimized. The solution is stored in parameters $\hat{y}_i^H$ and $\hat{y}_i^S$.

Finally, the CHHs locations selected by $\mathcal {P}^F_2$ are retained, and problem $\mathcal {P}_2$ is solved to refine the solution. It is based on the original objective function (22), retains the CHHs locations, and optimizes service activation, dimensioning and districting decisions:

$$\begin{aligned} \mathcal {P}_2: \left\{ \begin{array}{l} \min \; (22) \\ \text {s.t.} \\ \quad (1) - (21) \\ \quad y_i^H \le \hat{y}^H_i \qquad \forall i \in I \\ \quad y_i^S \le \hat{y}^S_i \qquad \forall i \in I \\ \end{array} \right. \end{aligned}$$

In $H_3$, feasibility is sought in a different way. It sets a maximum number $\kappa$ of changes with respect to the solutions obtained for the subdivisions and searches for the best solution within this distance by solving problem $\mathcal {P}_3$:

$$\begin{aligned} & \mathcal {P}_3: \\&\left\{ \begin{array}{l} \min \; (22) \\ \text {s.t.} \\ \quad (1)-(21) \\ \quad \sum \limits _{\begin{array}{l} i \in I: \\ \hat{y}_i^H = 0 \end{array}} y_i^H + \sum \limits _{\begin{array}{l} i \in I: \\ \hat{y}_i^H = 1 \end{array}} \left( 1-y_i^H \right) + \sum \limits _{\begin{array}{l} i \in I: \\ \hat{y}_i^S = 0 \end{array}}y_i^S + \sum \limits _{\begin{array}{l} i \in I: \\ \hat{y}_i^S = 1 \end{array}} \left( 1-y_i^S \right) \le \kappa \end{array} \right. \end{aligned}$$

Parameter $\kappa$ is set to a fraction of the number of candidate sites, i.e., $\kappa = \lceil |I|/\theta \rceil$. Obviously, if the value of $\kappa$ is too small, $H_3$ may not be able to find a feasible solution for the whole problem, while $H_1$ and $H_2$ are guaranteed to always find one.

### Additional steps

Two additional steps can be applied to each constructive heuristic solution. The solutions provided by the three heuristics can be either refined through a *k*-opt neighborhood search (*k*-opt) [42], or be used as warm start (WS) to solve the exact formulation $\mathcal {P}$ [41].

The *k*-opt neighborhood search is based on $\mathcal {P}_3$. The solution provided by $H_1$, $H_2$ or $H_3$ is stored in $\hat{y}^H_i$ and $\hat{y}^S_i$ and used as the initial solution, before $\mathcal {P}_3$ is iteratively solved. In a classic steepest descent local search framework, if the obtained objective function improves upon the best solution known so far, it becomes the new current best one, and $\mathcal {P}_3$ is solved again. Otherwise, the search stops.

Combining the constructive heuristics and additional steps, 12 different approaches are obtained, as reported in Table 3. It is worth mentioning that the approaches involving the WS provide both an upper and a lower bound for the exact $\mathcal {P}$ and can prove the optimality of the obtained solution. On the contrary, the others are purely heuristic and cannot guarantee optimality.

**Table 3:** Summary of the alternative approaches

Approach	Description
$H_1$	constructive heuristic $H_1$
$H_2$	constructive heuristic $H_2$
$H_3$	constructive heuristic $H_3$
$H_1$ + WS	$H_1$ solution used as WS for $\mathcal {P}$
$H_2$ + WS	$H_2$ solution used as WS for $\mathcal {P}$
$H_3$ + WS	$H_3$ solution used as WS for $\mathcal {P}$
$H_1$ + *k*-opt	$H_1$ followed by *k*-opt neighborhood search
$H_2$ + *k*-opt	$H_2$ followed by *k*-opt neighborhood search
$H_3$ + *k*-opt	$H_3$ followed by *k*-opt neighborhood search
$H_1$ + *k*-opt + WS	$H_1$ + *k*-opt solution used as WS for $\mathcal {P}$
$H_2$ + *k*-opt + WS	$H_2$ + *k*-opt solution used as WS for $\mathcal {P}$
$H_3$ + *k*-opt + WS	$H_3$ + *k*-opt solution used as WS for $\mathcal {P}$

## Computational tests

We first tested the DSS on three sets of synthetic instances with |*I*| equal to 100, 200 and 300, respectively (Sections 6.1 and 6.2). Then, we applied it to a relevant real-life case in Northern Italy (Sections 6.3 and 6.3.1).

We set parameter $\gamma$ to 20 for $H_2$ and $H_3$ in the synthetic instances, while clusters were directly taken from administrative data in the real-life case, and we set parameter $\theta$ to 20 in all tests. Moreover, we set the time limit (TL) for solving the formulation $\mathcal {P}$, as described in Section 4, to 3’600 seconds and for the others as reported in Table 4.

The approaches have been coded using gurobipy [24] and run in a jupyter environment on a computer equipped with an Intel^®^ Core i5-1135G7 CPU and 16 GB of RAM, running a Microsoft Windows 11 operating system. Geographical decomposition into subdivisions was done using the KMeans function from the Python package sklearn.

**Table 4:** TLs set for the heuristic approaches

Approach	Step	TL [s]
$H_1$	solving $\mathcal {P}^H$	360
	solving $\mathcal {P^M}$	360
	solving $\mathcal {P}_1$	360
$H_2$	solving $\mathcal {P}$ for each subdivision	180 each
	solving $\mathcal {P}^F_2$	900
	solving $\mathcal {P}_2$	900
$H_3$	solving $\mathcal {P}$ for each subdivision	180 each
	solving $\mathcal {P}_3$	900
Additional steps	*k*-opt	1’800 overall and 720 per iteration
	WS	1’800

### Synthetic instances

The considered cardinalities |*I*|, equal to 100, 200 and 300, are consistent with the size of the Italian provinces. According to the limits reported in Section 2, municipalities with less than 35’000 inhabitants can be considered a single territorial unit – or even the smallest and closest can be merged – while municipalities with more than 35’000 inhabitants must be divided into neighborhoods representing units $i \in I$ with less than 35’000 inhabitants. In Italy, the largest provinces by number of municipalities have about 300 municipalities, while the median value of municipalities for each province is 60 [25]. About 76% of the provinces have up to 100 municipalities, about 42% have 50 at most, about 20% have between 100 and 200, and only four out of 107 have more than 200, with the highest having 312. Although the larger cities need to be divided into units of less than 35’000 inhabitants, these statistics still prove the broad applicability of our approach.

For each |*I*|, we randomly generated the locations of the units over the area: for half of the instances, they were generated according to a uniform distribution, whereas a Gaussian distribution was used for in the other half. The population of each unit was also generated randomly based on either a lognormal or uniform distribution. Lognormal instances have a smaller population than the uniform ones, but the population range per unit is wider. The population per unit ranges from a few hundred to 35’000 for the lognormal instances, while, for the uniform instances, it ranges from 25’000 to 35’000 for $|I|=100$ and from 10’000 to 35’000 for $|I|=200$ and 300. The lognormal instances represent regions with many small villages and a few towns, while the uniform ones represent densely populated and urbanized areas, divided into neighborhoods. We also set two combinations for $\zeta _j$ and $\zeta _j^H$: 25 and 35 minutes or 35 and 45 minutes, respectively.

For each combination of |*I*|, choices of distribution and set of $\zeta _j$ and $\zeta _j^H$ parameters, we generated three instances: two with a square area of about 9’000 $km^2$ and one with a rectangular area of about 4’500 $km^2$, which is consistent with the Italian setting.

Finally, for the synthetic instances, travel times were calculated based on the location of the units and assuming an average speed of 50 *km*/*h*.

The values of reference population $b^{min}$ and $b^{max}$ were set to 40’000 and 50’000, respectively, based on the reference population values presented in Table 1.

We considered four services: *i*) 24/7 care in $S^H$, *ii*) GPs’ assistance in $S^M \setminus S^H$, *iii*) nurses’ assistance in $S^M \setminus S^H$, *iv*) a demand-based service in $S \setminus S^M$. For the 24/7 care, $l^H_s$ was set to 100’000, while for GPs’ and nurses’ assistance, $l^M_s$ was set to 50’000 and 35’000, respectively. As for the demand-based service $S \setminus S^M$, we set $\alpha$ to 12 and the $d_{si}$ values were calculated as a function of the respective population $p_i$.

Although the exact cost of CHHs are difficult to determine, it is reasonable to assume that the cost of opening and equipping a CHH is much higher than its running costs, and that the cost of a hub CHH is higher than that of a spoke CHH. Therefore, we set $k^H = 1.25 \cdot k^S$ for each possible CHH location, $f_s = 0.005 \cdot k^S$, and the cost $c_s$ of one shift of service $s \in S$ (for one year) to $\frac{1}{5} f_s$. We set $\eta$ to 2, to ensure that each site can be assigned to a maximum of one hub and one spoke CHH.

Taking $\gamma = 20$, we obtained five clusters with $|I|=100$, 10 with $|I|=200$, 15 with $|I|=300$. Moreover, taking $\theta = 20$, we allow for solutions with up to $\kappa = 5$ changes for $|I|=100$, $\kappa = 10$ changes for $|I|=200$ and $\kappa = 15$ for $|I|=300$ using heuristic $H_3$.

A detailed summary of the features of the tested instances can be found in Appendix A.

### Results for the synthetic instances

We compare the results obtained by solving the formulation $\mathcal {P}$, as presented in Section 4 with those of the 12 approaches summarized in Table 3. In the following, the formulation of Section 4 will simply be denoted by $\mathcal {P}$, while the other approaches will be denoted by the names given to them in Table 3. As aforementioned, half of these 12 approaches are exact (as is $\mathcal {P}$) due to the WS, wherease the others are purely heuristic; thus, we have analyzed seven exact and six heuristic approaches.

Exact and heuristic approaches are analyzed separately: Tables 5, 6 and 7 report the aggregated results of the exact approaches, while Tables 8, 9 and 10 present those of the heuristic approaches.

**Table 5:** Comparison of exact approaches ($|I|=100$)

		${H_1}$		${H_2}$		${H_3}$	
	$\mathcal {P}$	WS	*k*-opt + WS	WS	*k*-opt + WS	WS	*k*-opt + WS
No. of UB found	24	24	24	24	24	24	24
No. of optima found	1	0	0	0	0	0	1
Avg. internal gap	0.46%	0.47%	0.38%	0.42%	0.32%	0.34%	0.35%
Max internal gap	2.34%	1.67%	1.41%	1.76%	1.18%	1.24%	1.42%
No. of best LB found	19	0	9	1	0	1	0
Avg. best LB gap	0.46%	0.45%	0.36%	0.40%	0.31%	0.32%	0.34%
Max best LB gap	2.31%	1.64%	1.41%	1.74%	1.16%	1.21%	1.40%
No. of best UB found	5	4	4	7	7	8	7
Avg. best UB gap	0.19%	0.18%	0.09%	0.13%	0.04%	0.05%	0.07%
Max best UB gap	1.46%	0.81%	0.57%	1.02%	0.44%	0.50%	0.68%
Avg. CPU time [*s*]	TL	1’901.58	2’610.50	1’981.67	2’489.82	2’144.58	2’587.72
Max CPU time [*s*]	TL	2’184.60	2’904.61	2’214.42	2’922.92	2’965.85	TL

**Table 6:** Comparison of exact approaches ($|I|=200$)

		$H_1$		$H_2$		$H_3$	
	$\mathcal {P}$	WS	*k*-opt + WS	WS	*k*-opt + WS	WS	*k*-opt + WS
No. of UB found	14	24	24	24	24	16	16
No. of optima found	0	0	0	0	0	0	0
Avg. internal gap	1.13%	0.82%	0.76%	0.64%	0.62%	0.40%	0.37%
Max internal gap	11.89%	2.78%	2.78%	1.72%	1.72%	1.69%	1.55%
No. of best LB found	9	12	16	9	8	2	1
Avg. best LB gap	1.13%	0.82%	0.76%	0.64%	0.61%	0.39%	0.37%
Max best LB gap	11.89%	2.78%	2.78%	1.72%	1.72%	1.68%	1.54%
No. of best UB found	7	2	3	4	11	1	3
Avg. best UB gap	0.88%	0.24%	0.18%	0.06%	0.04%	0.06%	0.04%
Max best LB gap	10.40%	1.27%	1.27%	0.26%	0.26%	0.34%	0.19%
Avg. CPU time [*s*]	TL	2’453.11	3’173.15	2’634.73	3’354.78	2’439.73	3’245.35
Max CPU time [*s*]	TL	2’880.17	3’600.23	3’738.04	4’458.10	3’105.57	3’825.61

**Table 7:** Comparison of exact approaches ($|I|=300$)

		$H_1$		$H_2$		$H_3$	
	$\mathcal {P}$	WS	*k*-opt + WS	WS	*k*-opt + WS	WS	*k*-opt + WS
No. of UB found	10	19	19	20	20	10	10
No. of optima found	0	0	0	0	0	0	0
Avg. internal gap	0.19%	1.22%	1.19%	0.56%	0.55%	0.20%	0.19%
Max internal gap	0.38%	5.48%	5.37%	1.92%	1.92%	0.29%	0.28%
No. of LB found	22	15	15	15	15	10	0
No. of best LB found	16	0	10	5	5	1	0
Avg. best LB gap	0.19%	1.23%	1.20%	0.66%	0.65%	0.19%	0.18%
Max best LB gap	0.38%	5.48%	5.37%	1.92%	1.92%	0.27%	0.28%
No. of best UB found	1	0	0	10	17	0	2
Avg. best UB gap	0.02%	0.63%	0.60%	0.02%	0.01%	0.03%	0.02%
Max best UB gap	0.11%	4.41%	4.30%	0.08%	0.06%	0.05%	0.06%
Avg. CPU time [*s*]	TL	2’582.97	3’302.89	3’457.04	4’177.14	2’831.91	3’551.99
Max CPU time [*s*]	TL	2’880.24	3’600.27	4’500.72	5’221.85	3’054.21	3’774.26

**Table 8:** Comparison of heuristic approaches ($|I|=100$)

	$H_1$	$H_1$ + *k*-opt	$H_2$	$H_2$ + *k*-opt	$H_3$	$H_3$ + *k*-opt
No. of UB found	24	24	24	24	24	24
Avg. best LB gap	0.93%	0.45%	0.72%	0.36%	0.50%	0.40%
Max best LB gap	3.17%	1.41%	2.22%	1.16%	1.88%	1.40%
No. of best UB found	0	0	0	1	2	3
Avg. best UB gap	0.66%	0.18%	0.45%	0.09%	0.23%	0.13%
Max best UB gap	2.39%	0.57%	1.50%	0.44%	1.16%	0.68%
Avg. CPU time [*s*]	101.55	810.47	181.63	689.79	344.54	812.15
Max CPU time [*s*]	384.57	1’104.59	414.31	1’122.83	1’165.81	1’885.84

**Table 9:** Comparison of heuristic approaches ($|I|=200$)

	$H_1$	$H_1$ + *k*-opt	$H_2$	$H_2$ + *k*-opt	$H_3$	$H_3$ + *k*-opt
No. of UB found	24	24	24	24	16	16
Avg. best LB gap	1.66%	0.85%	0.90%	0.63%	0.56%	0.44%
Max best LB gap	3.19%	2.78%	1.98%	1.72%	1.68%	1.54%
No. of best UB found	0	3	0	6	0	2
Avg. best UB gap	1.08%	0.27%	0.32%	0.05%	0.22%	0.11%
Max best UB gap	1.73%	1.27%	0.92%	0.26%	0.51%	0.31%
Avg. CPU time [*s*]	653.06	1’373.10	834.68	1’554.73	834.68	1’445.29
Max CPU time [*s*]	1’080.12	1’800.17	1’937.98	2’658.04	1’937.98	2’025.56

**Table 10:** Comparison of heuristic approaches ($|I|=300$)

	$H_1$	$H_1$ + *k*-opt	$H_2$	$H_2$ + *k*-opt	$H_3$	$H_3$ + *k*-opt
No. of UB found	19	19	20	20	10	10
Avg. best LB gap	1.96%	1.28%	0.85%	0.66%	0.37%	0.25
Max best LB gap	6.26%	5.37%	2.18%	1.92%	0.66%	0.42%
No. of best UB found	0	0	2	12	0	0
Avg. best UB gap	1.35%	0.67%	0.20%	0.01%	0.21%	0.09%
Max best UB gap	5.18%	4.30%	0.48%	0.10%	0.44%	0.20%
Avg. CPU time [*s*]	782.71	1’502.80	1’656.90	2’377.05	1’031.82	1’751.91
CPU time max[s]	1’080.17	1’800.22	2’700.60	3’421.80	1’254.16	1’974.20

The exact approaches are evaluated in terms of the total number of upper bounds found out of the 24 instances for each |*I*|, optimal solutions found, final gap, lower and upper bound, and CPU time. Regarding heuristics, we analyze the upper bound, the total number of instances for which a feasible solution was found out of the 24 instances for each |*I*|, the gaps from the best lower and upper bound, and the CPU time.^2^

**Fig. 2:**
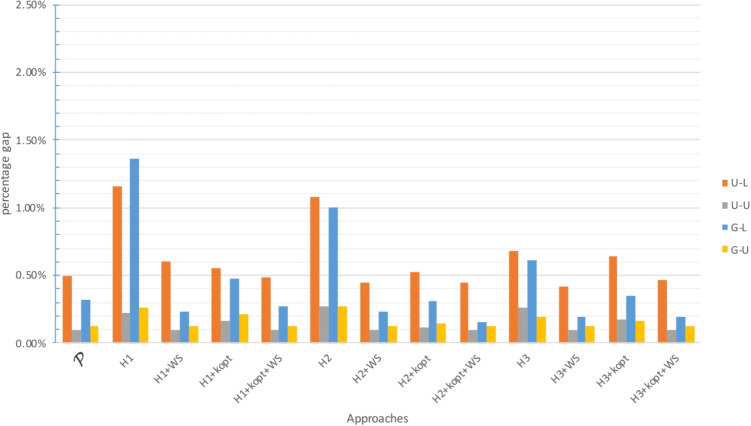
Gap with respect to the best known LB for $|I|=100$ with small travel threshold

**Fig. 3:**
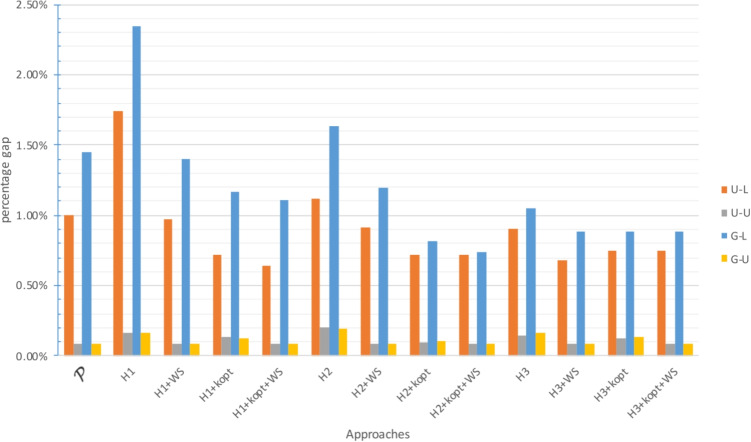
Gap with respect to the best known LB for $|I|=100$ with large travel threshold

**Fig. 4:**
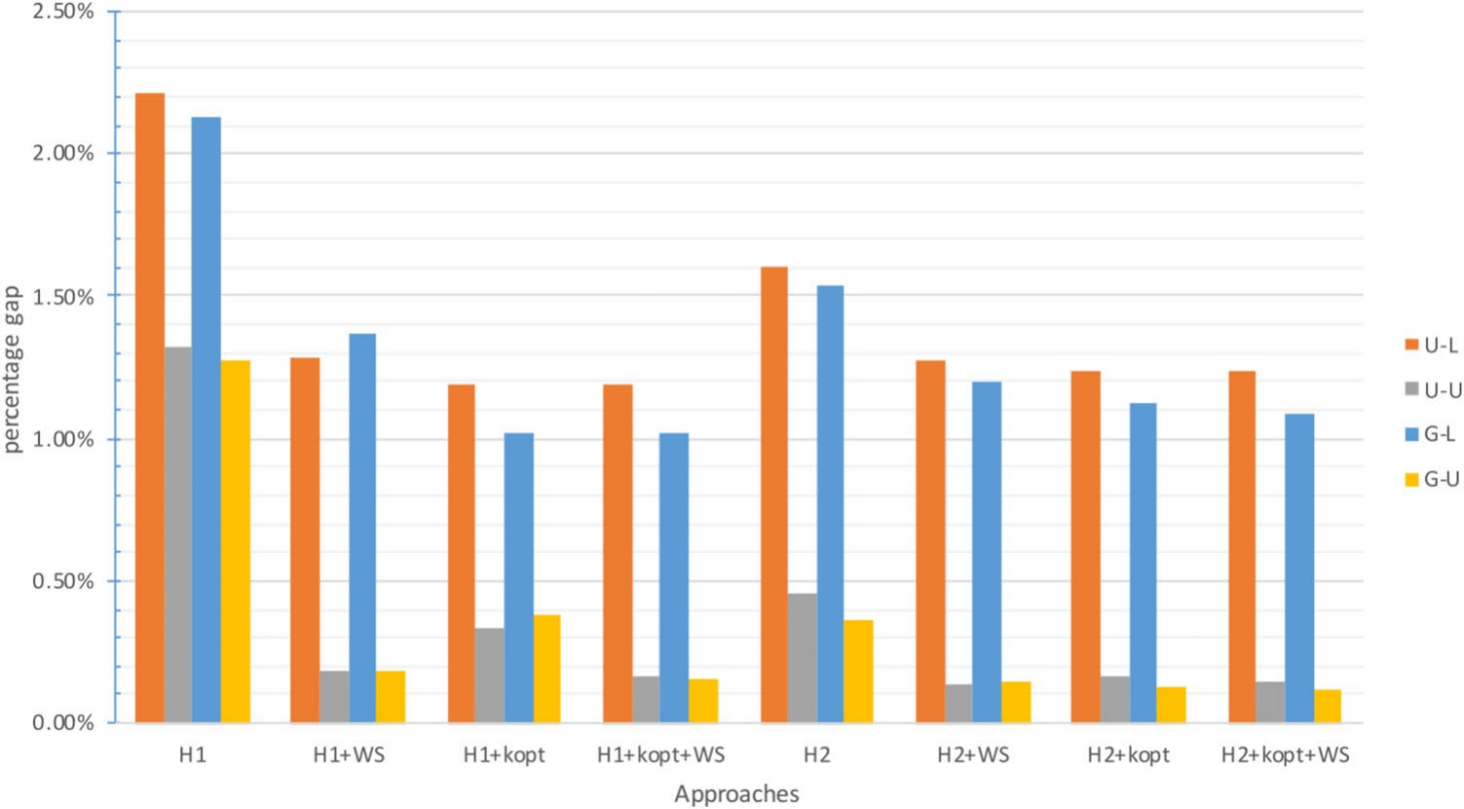
Gap with respect to the best known LB for $|I|=200$ with small travel threshold

**Fig. 5:**
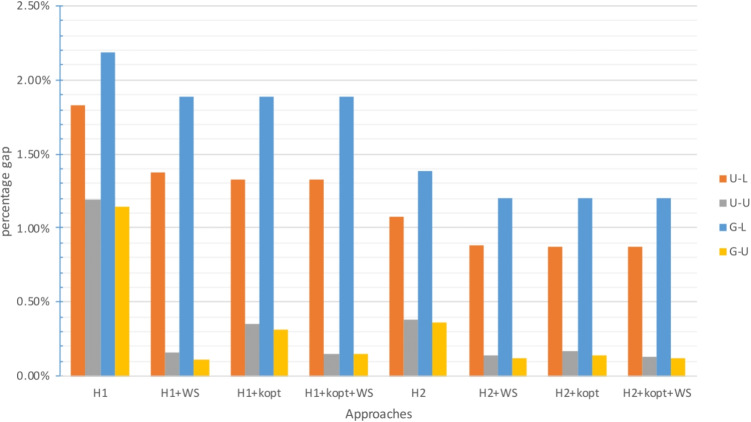
Gap with respect to the best known LB for $|I|=200$ with large travel threshold

**Fig. 6:**
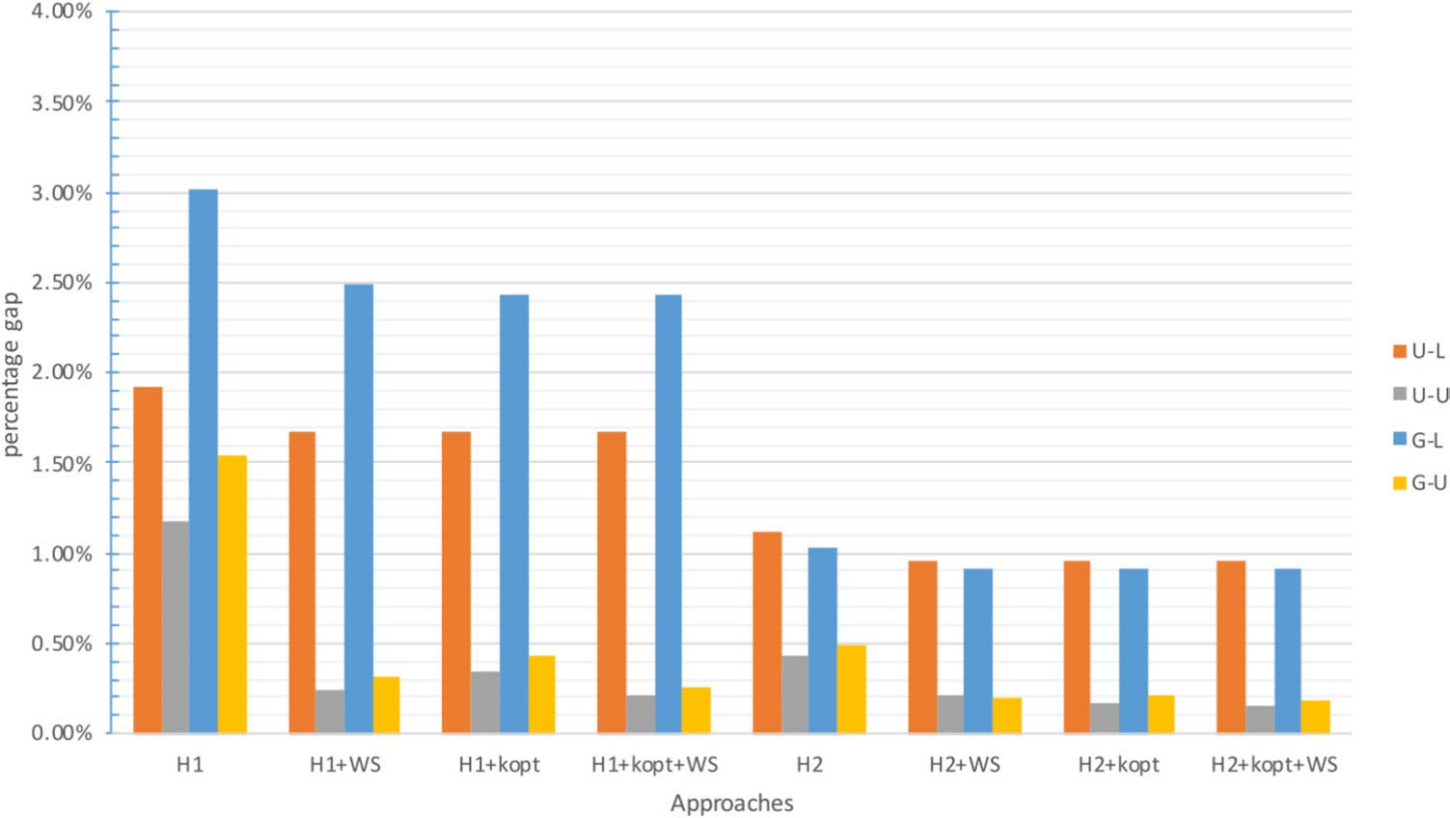
Gap with respect to the best known LB for $|I|=300$ with small travel threshold

**Fig. 7:**
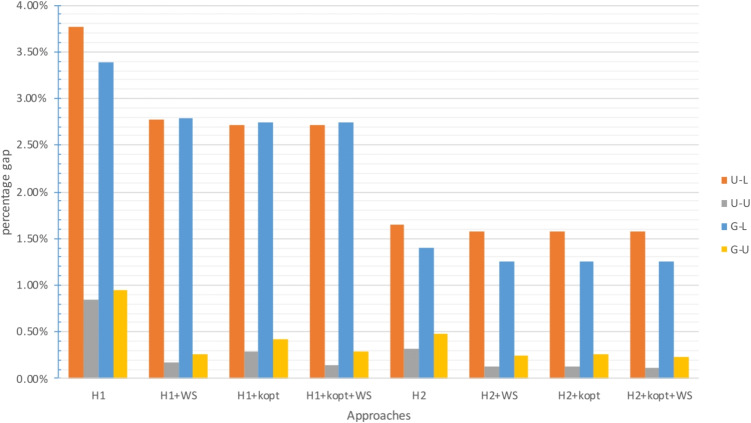
Gap with respect to the best known LB for $|I|=300$ with large travel threshold

The gap between the upper bound provided by the approach and the best known lower bound is analyzed in Figs. 2, 4 and 6 for $\zeta _j = 25$ and $\zeta ^H_j = 35$, and in Figs. 3, 5 and 7 for $\zeta _j = 35$ and $\zeta ^H_j = 45$. In these figures, instances are grouped based on the distribution used to generate territory positions and populations. The first letter denotes the distribution used for locations (U for uniform and G for Gaussian), while the second letter indicates the distribution used for populations (U for uniform and L for lognormal).

Obviously, the performance of the different approaches was affected by the size of the instance and the distributions employed. However, approaches based on heuristic $H_2$ generally provided better results and, among them, the best results were provided by $H_2$ + *k*-opt + WS. In fact, although it was not always the best approach, it provided stable and consistent results across all instances.

#### Results for the small instances

Tables 5 and 8 and Figs. 2 and 3 illustrate that all the approaches can successfully handle the instances with $|I|=100$. The performance of all approaches, whether exact or heuristic, is consistent and satisfactory.

Although only two instances were solved to optimality, all approaches produced excellent results. Their average percent gap from the best lower bound was always less than 0.5%, except for $H_1$, which had an average gap of 0.66%. The maximum gap was higher for the heuristic approaches but never exceeded 3.5%. The computational times were also reasonable. The heuristics were obviously faster, with a maximum computational time equal to half that of the exact approach. For all approaches, the U-L and G-L groups proved to be more challenging, with significantly larger gaps, generally about twice that of the U-U and G-U groups.

Although this result may vary depending on the quality of the lower and upper bounds found, it appears that the lognormal population distribution is more difficult to handle. Instances with large travel thresholds perform worse as far as the maximum gap is considered. In summary, all approaches can be used for all instances. The most promising one was $H_2$ + *k*-opt + WS, while $\mathcal {P}$ provided the worst performance and required the highest computational time (Figs. 4 and 5).

#### Results for the medium-size instances

Instances $|I|=200$ proved more difficult to solve. $\mathcal {P}$ failed to find a feasible solution for 10 out of 24 instances, while the exact methods based on $H_3$ failed to find a feasible solution for 8 out of 24 instances^3^, although they provided excellent results when they found a feasible solution.

Approaches based on $H_1$ and $H_2$ (both exact and heuristic) always yielded a feasible solution. $H_2$-based approaches provided the best results and an average gap from the best lower bound of 1% at most. In particular, the $H_2$-based approaches outperformed the $H_1$-based ones, while the $H_1$-based methods seemed to benefit the most from the final exact step. Although the gaps and computational times increased compared to the case with $|I|=100$, the $H_1$- and $H_2$-based approaches provided good results in a reasonable amount of time, and $H_2$ + *k*-opt was confirmed as the best approach with or without ($H_2$ + *k*-opt + WS) final exact step.

As observed in the instances with $|I|=100$, the lognormal population distribution and large travel thresholds led to the most challenging instances. However, even in these cases, the gap never exceeded 3%.

#### Results for the large instances

The previously identified trend is further confirmed for the instances with $|I|=300$. The number of instances in which an upper bound can be found decreased, with no approach being able to find a feasible solution for 4 out of 24 instances.^4^

$\mathcal {P}$ and the $H_3$-based approaches struggled the most and could yield a feasible solution only for 10 out of 24 instances. For two instances, solving $\mathcal {P}_3$ did not even produce a lower bound. The $H_2$-based approaches clearly outperformed the $H_1$-based ones, whether exact or heuristic. Despite the increase in gaps and computational times compared to the instances with $|I|=200$, the $H_2$-based approaches provided good results: the average gap was less than 1% for all these approaches and never exceeded 2.5%. The $H_3$-based approaches were able to provide very good results as well when they succeeded in solving instances, but they were not reliable.

The G-L and U-L instances were the most challenging: not only were they associated with the highest gaps, as shown in Figs. 6 and 7, but $\mathcal {P}$ and the $H_3$-based approaches could not even find a feasible solution for them.

### Real-life instance

In addition to testing on synthetic data, we applied the DSS to a real-life scenario in the Lombardy Region, namely the case of the Province of Brescia. We specifically considered this case because this province offers a unique setting, being among the largest provinces in Italy and including a heterogeneous territory with urban districts, flatlands and mountain areas. Therefore, it represents a challenging setting whose dimensions are no less than those found throughout the Italian context.

**Fig. 8:**
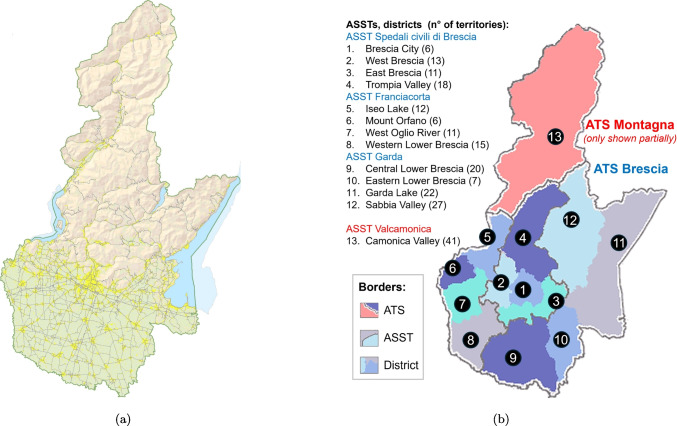
(a) Morphological map of the Province of Brescia displaying urban settlements (in yellow) and main roads (gray lines), obtained from the provincial cartography service (https://sit.provincia.brescia.it/download/cartografia-provinciale); (b) administrative subdivision of the province, including ATS, ASST and district level, taken from the data of the Lombardy Region (https://www.regione.lombardia.it/wps/portal/institutional/HP/institutional/Giunta/sedute-delibere-giunta-regionale/DettaglioDelibere/delibera-4563-legislatura-11)

According to the Italian National Statistics Institute and as of January 1^st^, 2024^5^, the Brescia Province ranks fourth in Italy by number of townships, with a total of 205, and fifth in terms of population, with 1,260,955 inhabitants. Its capital, the namesake city of Brescia, is the second biggest municipality in the Lombardy Region (after Milan), and the 16^th^ biggest nationally. A morphological map of the province, which indicates urban developments, is presented in Fig. 8a. The territory includes a large urban area around the capital, a vast flatland with some minor urban developments and a considerable number of small towns and villages, a sparsely inhabited mountainous area, and a densely inhabited lake shore. It is landlocked, but the presence of three lakes (the larger Lake Garda, Lake Iseo and the smaller Lake Idro) has allowed us to also incorporate into the analysis the unique strip-like population distribution that can be typically observed in coastal areas. Finally, the largest lake island in Italy (and southern Europe), called Monte Isola, can be found in Lake Iseo.

In terms of health services organization, the province is served by two Health Agencies called *Agenzie di Tutela della Salute* (ATSs): ATS Brescia and ATS Montagna. The ATS Brescia manages healthcare services in the central and southern areas of the province, including the city of Brescia, while the ATS Montagna covers the northern mountainous regions. The ATS Montagna also serves the Province of Sondrio and the northern part of the Province of Como. ATSs are responsible for the planning, coordination and management of healthcare services within their given territory as well as for public health initiatives, prevention programs and overall health governance, which includes monitoring the quality and accessibility of health services.

Three Territorial Social and Health Authorities, named *Agenzie Socio Sanitarie Territoriali* (ASSTs), operate in the ATS Brescia area (ASST Spedali Civili di Brescia, ASST Franciacorta, and ASST Garda), while one ASST (ASST Valcamonica) operates in the ATS Montagna area. They act as service providers in their reference territories, delivering health and social services directly to the population, and they encompass hospitals, outpatient clinics and other facilities where patients receive care.

Finally, each ASST is subdivided into districts, as shown in Fig. 8b. The division in districts depends on specific geographical areas that define the scope of their service provision, ensuring that health and social services are accessible and adapted to the needs of the municipalities.

We considered each municipality to be a territorial unit. Only the city of Brescia was divided it into five units according to the subdivision currently used for statistical purposes di the aforementioned National Statistics Institute. In this way, we obtained $|I|=209$, the coordinates for each of which were taken as the center of their respective municipality, which is a good proxy for the center of mass of the population distribution within each territory. For the city of Brescia, the city hall was used to locate the most central unit, while the decentralized city hall zonal offices were used to determine the locations of the other four units. These data were retrieved from the OpenStreetMap repository [7].

Data on the resident population and healthcare needs were obtained from administrative data provided by the Lombardy Region retrospectively using historical data on the medical services provided for the population by the regional healthcare service. We used data from the year 2018, as it was the most recent year for which there was no impact of the COVID-19 pandemic. We analyzed a database of 17,200,275 outpatient services provided in that year. To avoid overfitting, we consolidated the data into a set of higher-level services similar to those described in Fig. 1.

We then proceeded to the quantify the services considered in this real-life application. For $S^H$, we quantified the need for 24/7 care based only on the size of the resident population. A similar reasoning was adopted for the estimation of services in $S^M\setminus S^H$, i.e., GP and nursing services. For $S\setminus S^M$, we quantified the services provided to residents based not only on the size of the population but also on the historical number of services provided in their residence district. We initially considered all historically-provided specialties, that is cardiology, dermatology, echography, endocrinology, gastroenterology, neurology, neuropsychiatry, obstetrics and gynecology, oncology, ophthalmology, orthopedics, otorhinolaryngology, physical therapy, pneumology and urology. Next, we computed the total amount of these services provided in each district, which were then proportionally distributed to each territory contained in the district according to their resident population, which was taken from registry data. Finally, we included in $S \setminus S^M$ the most used services that account for half of the total services provided in the district; in other words, we included the most frequently provided service in the district, followed by the second most frequent, and so on, until we had accounted for 50% of the total services provided. This was done at the district level to account for local variability in the healthcare needs of the population. Accordingly, the services included in $S\setminus S^M$ were a district-specific combination of four or five of the following specialties: cardiology, echography, ophthalmology, orthopedics and physiotherapy.

As mentioned in Section 6.1, the exact costs associated to CHHs are difficult to determine, and we assumed a proportional hierarchy among them. In particular, the cost of opening a CHH ($k^H$, $k^S$) could vary enormously depending on whether the solution requires the construction of a new building *ex novo* or a simple conversion of already owned spaces. Specific data on available spaces and/or estimates of the construction work required, which are currently unavailable, are beyond the scope of this work.

While acknowledging that multiple modes of transport may be required for vulnerable populations, driving times were employed in this test, so as to balance simplicity and generalizability for different contexts. However, in very dense urban areas or areas with a limited number of cars, travel time would be better computed considering public transport and/or other relevant modes of travel. We argue that the Province of Brescia has neither.

A total of 13 clusters were considered, directly based on the administrative division, while $\theta = 20$ gave up to $\kappa = 11$ changes. Finally, we set $\zeta _j^H = 20$ and $\zeta _j = 30$. A single instance was prepared and used to test all methods.

#### Results for the real-life instance

Although this real-life instance includes $|I|=209$ territorial units, the results did not resemble those of the synthetic ones with $|I|=200$, and the instance behaved very differently due to its heterogeneity and proved difficult to solve. In fact, a heuristic approach was necessary because the exact method was unable to find a solution within the TL. The results of the heuristic approaches are reported in Table 11.

**Table 11:** Results for the real-life instance

	Time	Iterations	Gaps		
		for *k*-opt	Best UB	Internal LB	Best LB
$H_1$	TL		1.84%		6.57%
${H_1}$ + *k*-opt	TL	5	0.88%		5.56%
$H_2$	432.5 *s*		0.98%		5.67%
${H_2}$ + *k*-opt	TL	1	0.98%		5.67%
$H_3$	37.1 *s*		0.51%		5.18%
${H_3}$ + *k*-opt	TL	3	0.05%		4.70%
${H_1}$ + WS	TL		1.01%	5.65%	5.71%
${H_1}$ + *k*-opt + WS	TL		0.88%	5.56%	5.56%
${H_2}$ + WS	TL		0.98%	5.65%	5.67%
${H_2}$ + *k*-opt + WS	TL		0.98%	5.65%	5.67%
${H_3}$ + WS	TL		0.09%	4.69%	4.75%
${H_3}$ + *k*-opt + WS	TL		*	4.59%	4.65%

The best solution found is denoted by an asterisk, and gaps are reported rather than the actual values of the objective function, as the values of the coefficients for pricing ($k^H$, $k^S$, $f_s$ and $c_s$) are not relevant to the quality of the achievable solution, and only their ratio is. The computational gap with respect to the internal LB is reported for the exact methods only

All methods, except for the two constructive heuristics based on geographical decomposition, reached their respective TLs (set as indicated in Table 4). Comparing $H_2$ and $H_3$, the latter is faster, having been solved in less than a minute. For the methods using the *k*-opt, they usually stop in a maximum of five iterations because they cannot find an improving solutions nor prove that it does not exist within the TL assigned to a single iteration. The best solution was obtained by the WS applied to a *k*-opt algorithm constructed from the solution proposed by $H_3$ (i.e., $H_3$ + *k*-opt + WS). In contrast, the worst solution was found by $H_1$, which is similar to what was observed for the synthetic instances. The best solution itself showed a gap of 4.59% with respect to its internal lower bound and of 4.65% with respect to the best known lower bound of the solution, which was obtained during the computation of $H_1$ + *k*-opt + WS. Overall, the exact methods yielded an average internal gap of 5.30% and an average gap of 5.45% with respect to the best known lower bound.

**Fig. 9:**
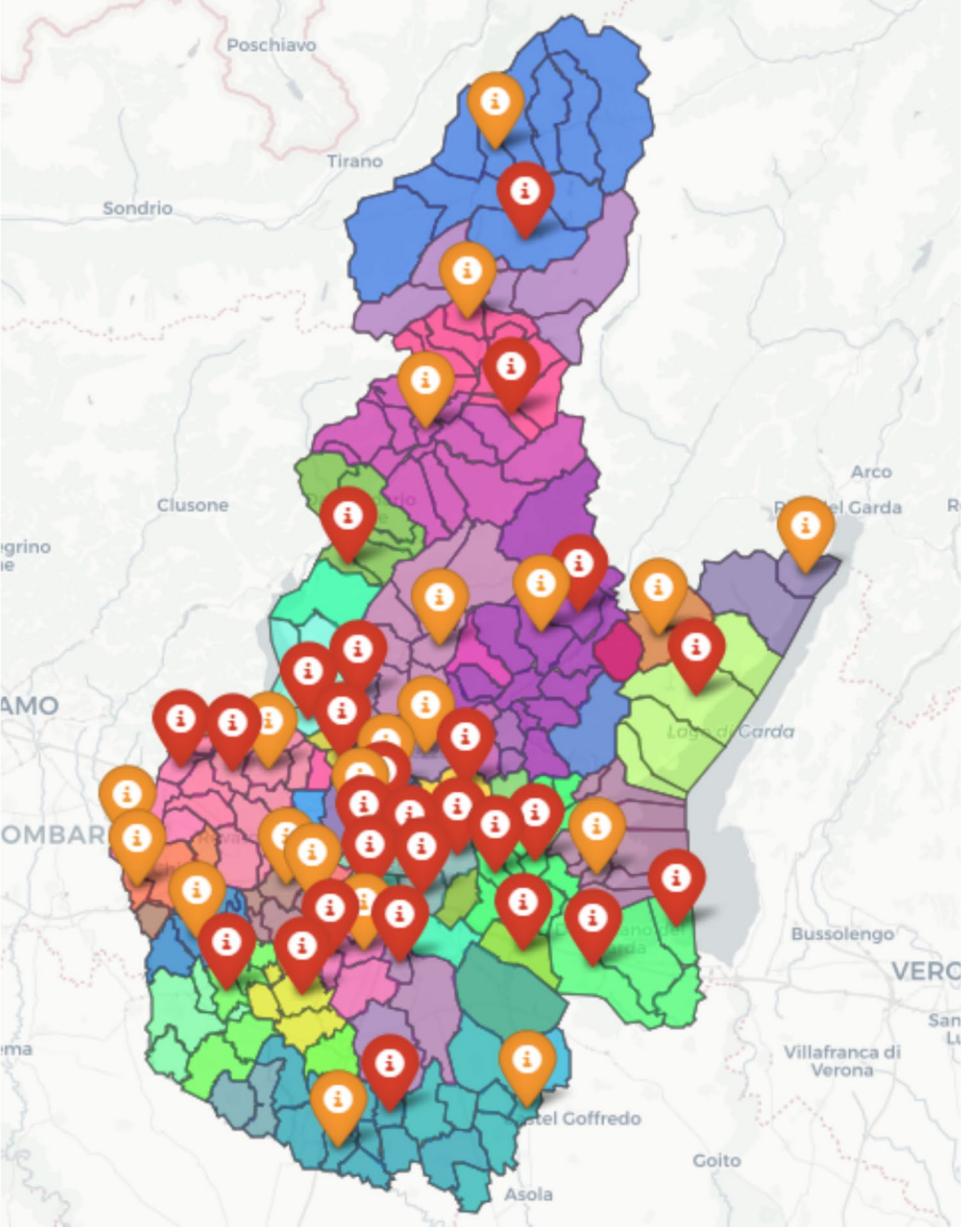
Screenshot of the visual interactive tool. The red markers denote the locations of hub CHHs, while the orange ones indicate the locations of spoke CHHs. The choropleth map highlights districting decisions for both types of CHH using transparent shading

To present the solutions in a compact manner, we developed a visual interactive tool that displays the CHHs locations and districts in their territory. This tool can be used by policymakers to increase their engagement in the decision-making process. Fig. 9 shows a screenshot of the tool, and the interactive version can be explored through the .html file provided in the Supplementary Materials to better understand the allocation of each territorial unit to a CHH.

## Discussion and conclusions

The DSS proposed in this paper can quantitatively support the design of an effective CHH network, including decisions about where to locate CHHs, what services to provide in each of them, and how to district the territories. The developed methodology, which is based on the proposed ILP model, ensures a thorough understanding and efficient management of the system under consideration at all levels, owing to its modular structure.

Although the features of the DSS are based on Italian law, it has general validity and may also be appropriate for other countries – at the very least in the European context. This is possible because the overall DSS structure is entirely parametrized and can be applied to any context of public healthcare service in which primary care is provided outside of hospitals and secondary care can be provided (at least in part) in the same facility. Therefore, decision-makers in other contexts could easily adapt the input parameters to their use case.

From a methodological standpoint, our formulation includes several features at the same time, e.g., simultaneous location, districting and dimensioning decisions for CHHs, accessibility and equity requirements, hub and spoke CHHs, population-based mandatory services and demand-based additional services.

We quantitatively considered equity according to the legal framework of our problem, which states that CHHs must serve a reference population of a predefined size regardless of the population density of the given territory. However, we quantified accessibility to the services in terms of travel times, binding them to the thresholds to strengthen equity. In fact, the reference population and travel time thresholds together provide equitable access in both urban and rural areas. On the one hand, the upper limit of the reference population prevents a single CHH from serving too many people in densely populated areas and, thereby, excessive crowding; on the other hand, appropriate travel time threshold values ensure that a CHH is available within a reasonable distance even in sparsely populated areas.

Thanks to the constructive heuristics, our DSS can effectively address real-sized and heterogeneous problem instances, such as that of the Province of Brescia presented in Section 6.3. This capability to handle larger problems may enable a more granular division of territories, further improving the customization of the services offered and their adherence to the healthcare needs of the population.

Heuristic $H_1$ also allowed us to test the effect of making decisions sequentially rather than simultaneously, i.e., the case in which the locations of the CHHs were first fixed, then the services they were to offer were decided, and, last the number of shifts were generated by allocating territories. The obtained results proved that, even if the simultaneous approach is more computationally demanding, it leads to better solutions.

The DSS can enable decision-makers to compare different policies comprehensively, while considering their impact on both service quality and cost. For example, it allows the assessment of the impact of the $\zeta _i^H$ and $\zeta _i$ values and it can also be used to assess whether the structure of the service provided is adequate from a workforce perspective, i.e., to check whether the number of scheduled shifts of a certain type aligns with contractual obligations. Finally, it can also serve as a resilient DSS that is capable of adapting to future changes. For instance, the model can be re-deployed with certain decisions, such as the location of CHHs fixed, in order to evaluate the introduction of new services or change the way in which they are offered.

### Assumptions, limitations and future development

We assume that assignments do not take user preferences into account, and users are assigned to the closest CHH for each service. Despite it, patients are expected to mostly choose their own referral center, which makes this assumption reasonable. Future work will be devoted to taking user preferences into account. For example, we could assume that a fraction of the population prefers the second closest CHH over the first and test whether the sizing is still appropriate. In addition, decisions made at higher levels, including strategic and tactical planning, will be integrated with operational decisions, such as staff recruitment and rostering. For instance, future work will extend the DSS to include a feedback loop between these decision levels.

In addition, although geographical barriers were considered using travel times and properly accounted for in the real-life instance, we considered only one mean of transportation, which may not be suitable for vulnerable populations. In the future, we will also include other means of transportation, specifying travel times according to individual user profiles.

Finally, we assumed all parameters to be deterministic. However, since uncertainty may play a role, we will extend the models toward stochastic or robust formulations to include, for example, uncertain service amounts or population choices.

As for costs, it is worth noting that we assumed a proportional hierarchy among them, as this was not a limitation in our experiments. However, for actual future applications, the exact costs will need to be considered to avoid bias in the solutions.

## Supplementary Information

Below is the link to the electronic supplementary material.

Supplementary file 1 (html 17973 KB)

## Data Availability

Data of the instances will be made available upon request.

## Appendix A: Instances features

Test instances were built by randomly generating distinctive characteristics of hypothetical territories/population combinations, that were capable of representing several real-life scenarios.

We assumed that the considered territory can be composed of $|I|=100$, 200 and 300 units. This is representative of most cases related to the Italian system wherein the largest province (Turinin the Piedmont Region) includes 312 municipalities, while the median value is 60.^6^

For two-thirds of the instances, the positions of the territorial units were generated within a square projected onto the surface of the Earth between 44° and 45° latitude and 9° and 10° longitude (instances of type “A” and “B”), for a total area of 8’818.29 $km^2$. For the remaining third, the positions were generated within a rectangle between 44° and 45° latitude and 9° and 9.5° longitude (instances of type “C”), for a total area of 4’409.23 $km^2$. Next, two alternative distributions were used to generate the positions: a Gaussian distribution, centered in the middle of the territory, or a uniform distribution. Finally, the distances between the units were measured using the Haversine formula and converted into travel times assuming a speed of 50 *km*/*h*.

Two distributions were also used to generate the population of each unit: a lognormal and a uniform distribution.

Further details are given in Tables 12, 13 and 14, for $|I|=100$, 200, and 300, respectively. The name of each instance is structured as I-*dim*-*loc*-*pop*-*group*-*travel*, where *dim* is the dimension of the instance given by |*I*|, *loc* is “U” (or “G”) depending on whether the locations of the units were generated with a uniform (or Gaussian) distribution, *pop* is “L” (or “U”) depending on whether the populations were generated with a lognormal (or uniform) distribution, *group* is “A”, “B”, or “C” which also defines the shape of the territory, and *travel* refers to the maximum travel times $\zeta _i$ and $\zeta _i^H$ and is denoted by “s” for short and “l” for long.

**Table 12:** List of instances with $|I|=100$

Name	Unit spatial	Shape	Area	Population	Population				
	distribution		[$km^2$]	distribution	Tot.	Min.	Max.	$\zeta _i$	$\zeta _i^H$
I-100-U-L-A-s	uniform	square	8’818.29	lognormal	692’641	317	35’000	25	35
I-100-U-L-B-s	uniform	square	8’818.29	lognormal	656’520	587	30’506	25	35
I-100-U-L-C-s	uniform	rectangular	4’409.23	lognormal	677’844	772	35’000	25	35
I-100-U-U-A-s	uniform	square	8’818.29	uniform	2’956’672	25’078	34’901	25	35
I-100-U-U-B-s	uniform	square	8’818.29	uniform	2’983’565	25’084	34’959	25	35
I-100-U-U-C-s	uniform	rectangular	4’409.23	uniform	3’009’776	25’047	34’972	25	35
I-100-G-L-A-s	Gaussian	square	8’818.29	lognormal	686’271	830	35’000	25	35
I-100-G-L-B-s	Gaussian	square	8’818.29	lognormal	702’661	1070	35’000	25	35
I-100-G-L-C-s	Gaussian	rectangular	4’409.23	lognormal	708’807	450	35’000	25	35
I-100-G-U-A-s	Gaussian	square	8’818.29	uniform	3’025’590	25’140	34’836	25	35
I-100-G-U-B-s	Gaussian	square	8’818.29	uniform	2’979’795	25’064	34’858	25	35
I-100-G-U-C-s	Gaussian	rectangular	4’409.23	uniform	3’032’501	25’082	34’964	25	35
I-100-U-L-A-l	uniform	square	8’818.29	lognormal	731’684	571	31’711	35	45
I-100-U-L-B-l	uniform	square	8’818.29	lognormal	766’492	895	35’000	35	45
I-100-U-L-C-l	uniform	rectangular	4’409.23	lognormal	706’085	672	35’000	35	45
I-100-U-U-A-l	uniform	square	8’818.29	uniform	3’021’133	25’070	34’870	35	45
I-100-U-U-B-l	uniform	square	8’818.29	uniform	2’996’003	25’043	34’897	35	45
I-100-U-U-C-l	uniform	rectangular	4’409.23	uniform	2’999’535	25’084	34’904	35	45
I-100-G-L-A-l	Gaussian	square	8’818.29	lognormal	722’862	813	35’000	35	45
I-100-G-L-B-l	Gaussian	square	8’818.29	lognormal	785’951	823	35’000	35	45
I-100-G-L-C-l	Gaussian	rectangular	4’409.23	lognormal	697’935	484	35’000	35	45
I-100-G-U-A-l	Gaussian	square	8’818.29	uniform	3’039’427	25’013	34’999	35	45
I-100-G-U-B-l	Gaussian	square	8’818.29	uniform	2’978’051	25’016	34’714	35	45
I-100-G-U-C-l	Gaussian	rectangular	4’409.23	uniform	3’005’350	25’061	34’990	35	45

**Table 13:** List of instances with $|I|=200$

Name	Unit spatial	Shape	Area	Population	Population				
	distribution		[$km^2$]	distribution	Tot.	Min.	Max.	$\zeta _i$	$\zeta _i^H$
I-200-U-L-A-s	uniform	square	8’818.29	lognormal	1’418’237	265	35’000	25	35
I-200-U-L-B-s	uniform	square	8’818.29	lognormal	1’454’966	435	35’000	25	35
I-200-U-L-C-s	uniform	rectangular	4’409.23	lognormal	1’479’882	655	35’000	25	35
I-200-U-U-A-s	uniform	square	8’818.29	uniform	4’447’116	10’252	34’779	25	35
I-200-U-U-B-s	uniform	square	8’818.29	uniform	4’582’369	10’226	34’917	25	35
I-200-U-U-C-s	uniform	rectangular	4’409.23	uniform	4’379’705	10’003	34’802	25	35
I-200-G-L-A-s	Gaussian	square	8’818.29	lognormal	1’466’210	390	35’000	25	35
I-200-G-L-B-s	Gaussian	square	8’818.29	lognormal	1’415’712	675	35’000	25	35
I-200-G-L-C-s	Gaussian	rectangular	4’409.23	lognormal	1’476’889	420	35’000	25	35
I-200-G-U-A-s	Gaussian	square	8’818.29	uniform	4’438’191	10’061	34’987	25	35
I-200-G-U-B-s	Gaussian	square	8’818.29	uniform	4’539’335	10’059	34’892	25	35
I-200-G-U-C-s	Gaussian	rectangular	4’409.23	uniform	4’489’774	10’103	34’972	25	35
I-200-U-L-A-l	uniform	square	8’818.29	lognormal	1’336’017	518	35’000	35	45
I-200-U-L-B-l	uniform	square	8’818.29	lognormal	1’502’323	562	35’000	35	45
I-200-U-L-C-l	uniform	rectangular	4’409.23	lognormal	1’339’998	436	35’000	35	45
I-200-U-U-A-l	uniform	square	8’818.29	uniform	4’445’356	10’312	34’794	35	45
I-200-U-U-B-l	uniform	square	8’818.29	uniform	4’432’893	10’058	34’687	35	45
I-200-U-U-C-l	uniform	rectangular	4’409.23	uniform	4’564’457	10’126	34’876	35	45
I-200-G-L-A-l	Gaussian	square	8’818.29	lognormal	1’287’516	545	35’000	35	45
I-200-G-L-B-l	Gaussian	square	8’818.29	lognormal	1’324’755	511	35’000	35	45
I-200-G-L-C-l	Gaussian	rectangular	4’409.23	lognormal	1’371’012	316	35’000	35	45
I-200-G-U-A-l	Gaussian	square	8’818.29	uniform	4’366’375	10’248	34’787	35	45
I-200-G-U-B-l	Gaussian	square	8’818.29	uniform	4’659’209	10’566	34’922	35	45
I-200-G-U-C-l	Gaussian	rectangular	4’409.23	uniform	4’370’317	10’003	34’669	35	45

**Table 14:** List of instances with $|I|=300$

Name	Unit spatial	Shape	Area	Population	Population				
	distribution		[$km^2$]	distribution	Tot.	Min.	Max.	$\zeta _i$	$\zeta _i^H$
I-300-U-L-A-s	uniform	square	8’818.29	lognormal	2’296’427	487	35’000	25	35
I-300-U-L-B-s	uniform	square	8’818.29	lognormal	2’2750’21	358	35’000	25	35
I-300-U-L-C-s	uniform	rectangular	4’409.23	lognormal	2’255’893	507	35’000	25	35
I-300-U-U-A-s	uniform	square	8’818.29	uniform	6’691’233	10’061	34’886	25	35
I-300-U-U-B-s	uniform	square	8’818.29	uniform	6’746’671	10’059	34’991	25	35
I-300-U-U-C-s	uniform	rectangular	4’409.23	uniform	6’707’655	10’044	34’993	25	35
I-300-G-L-A-s	Gaussian	square	8’818.29	lognormal	2’380’917	621	35’000	25	35
I-300-G-L-B-s	Gaussian	square	8’818.29	lognormal	2’135’249	160	35’000	25	35
I-300-G-L-C-s	Gaussian	rectangular	4’409.23	lognormal	2’152’682	281	35’000	25	35
I-300-G-U-A-s	Gaussian	square	8’818.29	uniform	6’528’161	10’090	34’920	25	35
I-300-G-U-B-s	Gaussian	square	8’818.29	uniform	6’603’085	10’027	34’908	25	35
I-300-G-U-C-s	Gaussian	rectangular	4’409.23	uniform	6’894’359	10’069	34’981	25	35
I-300-U-L-A-l	uniform	square	8’818.29	lognormal	2’195’042	382	35’000	35	45
I-300-U-L-B-l	uniform	square	8’818.29	lognormal	2’176’301	277	35’000	35	45
I-300-U-L-C-l	uniform	rectangular	4’409.23	lognormal	1’885’796	272	35’000	35	45
I-300-U-U-A-l	uniform	square	8’818.29	uniform	6’965’046	10’006	34’887	35	45
I-300-U-U-B-l	uniform	square	8’818.29	uniform	6’759’483	10’073	34’883	35	45
I-300-U-U-C-l	uniform	rectangular	4’409.23	uniform	6’765’528	10’009	34’980	35	45
I-300-G-L-A-l	Gaussian	square	8’818.29	lognormal	1’950’229	445	35’000	35	45
I-300-G-L-B-l	Gaussian	square	8’818.29	lognormal	2’275’512	433	35’000	35	45
I-300-G-L-C-l	Gaussian	rectangular	4’409.23	lognormal	1’950’943	435	31’179	35	45
I-300-G-U-A-l	Gaussian	square	8’818.29	uniform	6’647’825	10’086	34’962	35	45
I-300-G-U-B-l	Gaussian	square	8’818.29	uniform	6’701’396	10’051	34’985	35	45
I-300-G-U-C-l	Gaussian	rectangular	4’409.23	uniform	6’853’751	10’056	34’887	35	45

## Footnotes

1 Additional info (in Italian): https://www.welforum.it/le-case-della-comunita-in-lombardia-unindagine

2 The percentage gap between a feasible solution *H* and the best known upper bound *UB* is computed as $\frac{H-UB}{UB}*100$, while that for the best known lower bound *LB* is computed as $\frac{H-LB}{LB}*100$.

3 Results are averaged only for those the instances for which a feasible solution was found. In the Figures, the results for only those approaches that found a feasible solution for all instances have been reported.

4 Results are averaged only for those the instances for which a feasible solution was found. In the Figures, the results for only those approaches that found a feasible solution for the majority of the instances have been reported.

5 Additional info: https://demo.istat.it/
